# Bis(triphenyl­phospho­ranyl­idene)ammonium iodide

**DOI:** 10.1107/S1600536810000267

**Published:** 2010-01-09

**Authors:** Michael A. Beckett, Peter N. Horton, Michael B. Hursthouse, James L. Timmis

**Affiliations:** a School of Chemistry, University of Wales, Bangor LL57 2UW, Wales; b School of Chemistry, University of Southampton, Highfield, Southampton SO17 1BJ, England

## Abstract

The title compound, C_36_H_30_NP_2_^+^·I^−^, was obtained accidently from crystallization of a reaction mixture containing [(Ph_3_P)_2_N]OH and B(OH)_3_, which was contaminated with MeI. There are two independent [(Ph_3_P)_2_N]^+^ cations and two I^−^ anions within the asymmetric unit. The central PNP angles are non-linear [137.6 (2) and 134.4 (2)°] and the phenyl substituents on P centres adopt different conformations within these two cations.

## Related literature

For crystal structures containing the [(Ph_3_P)_2_N]^+^ cation, see: Glidewell & Holden (1982 ▶); Guzei *et al.* (2001 ▶); Handy *et al.* (1970 ▶); Kirtley *et al.* (1980 ▶); Lewis & Dance (2000 ▶); Seel *et al.* (1984 ▶, 1985 ▶); Tebbe & Krauss (1990 ▶); Weller *et al.* (1993 ▶).
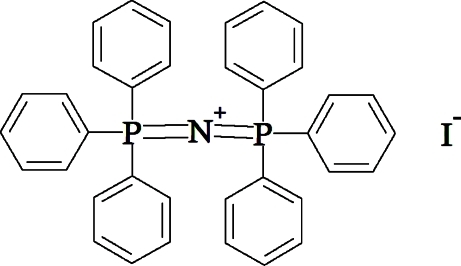


## Experimental

### 

#### Crystal data

C_36_H_30_NP_2_^+^·I^−^
*M* *_r_* = 665.45
Monoclinic, 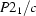

*a* = 29.6827 (6) Å
*b* = 10.1604 (2) Å
*c* = 20.2114 (4) Å
β = 91.337 (1)°
*V* = 6093.9 (2) Å^3^
*Z* = 8
Mo *K*α radiation
μ = 1.18 mm^−1^
*T* = 120 K
0.18 × 0.10 × 0.05 mm

#### Data collection

Bruker–Nonius APEXII CCD camera on κ-goniostat diffractometer
Absorption correction: multi-scan (*SADABS*; Sheldrick, 2007 ▶) *T*_min_ = 0.815, *T*_max_ = 0.943
52122 measured reflections
13900 independent reflections
11771 reflections with *I* > 2σ(*I*)
*R*_int_ = 0.054

#### Refinement

*R*[*F* ^2^ > 2σ(*F* ^2^)] = 0.048
*wR*(*F* ^2^) = 0.105
*S* = 1.11
13900 reflections
721 parameters
H-atom parameters constrained
Δρ_max_ = 0.64 e Å^−3^
Δρ_min_ = −0.59 e Å^−3^

### 

Data collection: *COLLECT* (Hooft, 1998 ▶); cell refinement: *DENZO* (Otwinowski & Minor, 1997 ▶) and *COLLECT*; data reduction: *DENZO* and *COLLECT*; program(s) used to solve structure: *SHELXS97* (Sheldrick, 2008 ▶); program(s) used to refine structure: *SHELXL97* (Sheldrick, 2008 ▶); molecular graphics: *ORTEP-3 for Windows* (Farrugia, 1997 ▶); software used to prepare material for publication: *WinGX* (Farrugia, 1999 ▶).

## Supplementary Material

Crystal structure: contains datablocks I, global. DOI: 10.1107/S1600536810000267/ci2990sup1.cif

Structure factors: contains datablocks I. DOI: 10.1107/S1600536810000267/ci2990Isup2.hkl

Additional supplementary materials:  crystallographic information; 3D view; checkCIF report

## Figures and Tables

**Fig. 1.**
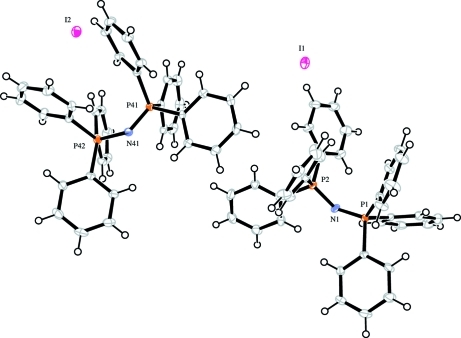
The asymmetric unit of the title compound. Displacement ellipsoids are drawn at the 50% probability level.

**Fig. 2.**
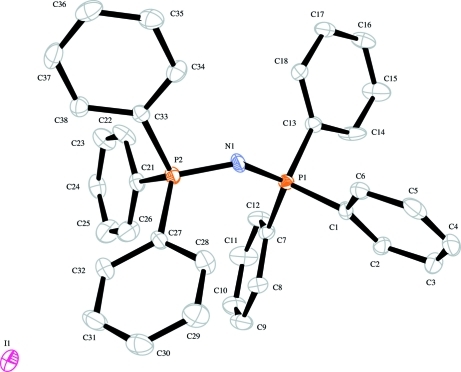
View of one of the two independent ionic units of the title compound, showing the atomic numbering scheme. Displacement ellipsoids are drawn at the 50% probability level.

## supplementary crystallographic information

### Comment

There are numerous reports of single-crystal structures containing the [(Ph_3_P)_2_N]^+^ cation (Lewis & Dance, 2000) and usually this cation is partnered by an equally bulky anion, *e.g.* [(Ph_3_P)_2_N][Cr_2_(CO)_10_I] (Handy *et al.*, 1970). Crystallographic studies on compounds containing [(Ph_3_P)_2_N]^+^ partnered with small anions are much more restricted *e.g.* [(Ph_3_P)_2_N][I_3_] (Tebbe & Krauss, 1990), [(Ph_3_P)_2_N][SCN] (Glidewell & Holden, 1982), [(Ph_3_P)_2_N][SNO_2_] (Seel *et al.*, 1984), [(Ph_3_P)_2_N][SNO].CH_3_COCH_3_ (Seel *et al.*, 1985), [(Ph_3_P)_2_N]Cl^.^PhCH_3_ (Weller *et al.*, 1993), and [(Ph_3_P)_2_N][HSO_4_].CHCl_3_ (Guzei *et al.*, 2001).

The unit cell of the title compound contains two independent [(Ph_3_P)_2_N]^+^ cations partnered by I^-^ anions, with no significantly close anion/cation contacts. The conformations of the phenyl groups and the PNP angles differ in the two cations present, and in both cases the PNP angles are at the low end of the generally observed range, 130–180° (Lewis & Dance, 2000). Independent cations with disparate angles 139.1° and 180° have been observed in [(Ph_3_P)_2_N]_3_{Na[Mo_3_(CO)_6_(NO)_3_(OCH_3_)_3_(O)]} (Kirtley *et al.*, 1980). The conformations of phenyl rings within the two cations are different with the cation containing N41 arranged so that there is an eclipsed interaction between phenyl rings of its two PPh_3 _moieties. This eclipsed arrangement is not apparent in the cation containing N1. Detailed analysis of inter- and intramolecular phenyl embraces within such cations has been described (Lewis & Dance, 2000).

### Experimental

A few crystals of [(Ph_3_P)_2_N] I, suitable for X-ray diffraction, were obtained from the crystallization of a reaction mixture containing [(Ph_3_P)_2_N][OH] (3.49 mmol) and B(OH)_3_ (1.08g, 17.43 mmol) in H_2_O/MeOH (15/15 cm^3^) which had been contaminated by MeI/ I^-^. MeI had been regularly used in the vicinity for methylation of amines.

### Refinement

H atoms were positioned geometrically [C-H = 0.95 Å] and refined using a riding model, with *U*_iso_(H) = 1.2*U*_eq_(C).

### Crystal data

**Table:**

C_36_H_30_NP_2_^+^·I^−^	*F*(000) = 2688
*M**_r_* = 665.45	*D*_x_ = 1.451 Mg m^−^^3^
Monoclinic, *P*2_1_/*c*	Mo *K*α radiation, λ = 0.71073 Å
Hall symbol: -P 2ybc	Cell parameters from 14269 reflections
*a* = 29.6827 (6) Å	θ = 2.9–27.5°
*b* = 10.1604 (2) Å	µ = 1.18 mm^−^^1^
*c* = 20.2114 (4) Å	*T* = 120 K
β = 91.337 (1)°	Slab, colourless
*V* = 6093.9 (2) Å^3^	0.18 × 0.10 × 0.05 mm
*Z* = 8	

### Data collection

**Table:**

Bruker–Nonius APEXII CCD camera on κ-goniostat diffractometer	13900 independent reflections
Radiation source: Bruker-Nonius FR591 rotating anode	11771 reflections with *I* > 2σ(*I*)
10cm confocal mirrors	*R*_int_ = 0.054
Detector resolution: 4096x4096pixels / 62x62mm pixels mm^-1^	θ_max_ = 27.5°, θ_min_ = 2.9°
φ and ω scans	*h* = −38→38
Absorption correction: multi-scan (*SADABS*; Sheldrick, 2007)	*k* = −13→13
*T*_min_ = 0.815, *T*_max_ = 0.943	*l* = −26→26
52122 measured reflections	

### Refinement

**Table:**

Refinement on *F*^2^	Primary atom site location: structure-invariant direct methods
Least-squares matrix: full	Secondary atom site location: difference Fourier map
*R*[*F*^2^ > 2σ(*F*^2^)] = 0.048	Hydrogen site location: inferred from neighbouring sites
*wR*(*F*^2^) = 0.105	H-atom parameters constrained
*S* = 1.11	*w* = 1/[σ^2^(*F*_o_^2^) + 23.1366*P*] where *P* = (*F*_o_^2^ + 2*F*_c_^2^)/3
13900 reflections	(Δ/σ)_max_ = 0.002
721 parameters	Δρ_max_ = 0.64 e Å^−^^3^
0 restraints	Δρ_min_ = −0.59 e Å^−^^3^

### Special details

**Table:**

Experimental. *SADABS* was used to perform the Absorption correction Parameter refinement on 49041 reflections reduced *R*(int) from 0.1282 to 0.0578 Ratio of minimum to maximum apparent transmission: 0.905943 The given Tmin and Tmax were generated using the *SHELX* SIZE command
Geometry. All e.s.d.'s (except the e.s.d. in the dihedral angle between two l.s. planes) are estimated using the full covariance matrix. The cell e.s.d.'s are taken into account individually in the estimation of e.s.d.'s in distances, angles and torsion angles; correlations between e.s.d.'s in cell parameters are only used when they are defined by crystal symmetry. An approximate (isotropic) treatment of cell e.s.d.'s is used for estimating e.s.d.'s involving l.s. planes.
Refinement. Refinement of *F*^2^ against ALL reflections. The weighted *R*-factor *wR* and goodness of fit *S* are based on *F*^2^, conventional *R*-factors *R* are based on *F*, with *F* set to zero for negative *F*^2^. The threshold expression of *F*^2^ > σ(*F*^2^) is used only for calculating *R*-factors(gt) *etc*. and is not relevant to the choice of reflections for refinement. *R*-factors based on *F*^2^ are statistically about twice as large as those based on *F*, and *R*- factors based on ALL data will be even larger.

### Fractional atomic coordinates and isotropic or equivalent isotropic displacement parameters (Å^2^)

**Table:**

	*x*	*y*	*z*	*U*_iso_*/*U*_eq_	
I1	0.369635 (8)	−0.03183 (2)	0.279022 (13)	0.02336 (7)	
I2	0.132635 (8)	0.79725 (2)	0.522407 (13)	0.02426 (7)	
C1	0.44904 (12)	0.1172 (4)	−0.13846 (17)	0.0166 (7)	
C2	0.48299 (12)	0.0265 (4)	−0.15317 (19)	0.0220 (8)	
H2	0.4791	−0.0644	−0.1438	0.026*	
C3	0.52246 (13)	0.0712 (5)	−0.1817 (2)	0.0296 (9)	
H3	0.5458	0.0108	−0.1914	0.036*	
C4	0.52758 (14)	0.2024 (5)	−0.1958 (2)	0.0313 (10)	
H4	0.5543	0.2320	−0.2161	0.038*	
C5	0.49427 (14)	0.2922 (4)	−0.1807 (2)	0.0287 (9)	
H5	0.4985	0.3830	−0.1898	0.034*	
C6	0.45437 (12)	0.2496 (4)	−0.15225 (18)	0.0205 (8)	
H6	0.4312	0.3107	−0.1425	0.025*	
C7	0.40561 (12)	−0.0902 (3)	−0.06631 (18)	0.0168 (7)	
C8	0.43838 (12)	−0.0866 (4)	−0.01529 (19)	0.0211 (8)	
H8	0.4550	−0.0082	−0.0071	0.025*	
C9	0.44652 (14)	−0.1973 (4)	0.0233 (2)	0.0279 (9)	
H9	0.4687	−0.1946	0.0579	0.033*	
C10	0.42216 (15)	−0.3126 (4)	0.0115 (2)	0.0320 (10)	
H10	0.4276	−0.3880	0.0383	0.038*	
C11	0.39005 (16)	−0.3173 (4)	−0.0393 (2)	0.0367 (11)	
H11	0.3736	−0.3961	−0.0474	0.044*	
C12	0.38185 (14)	−0.2067 (4)	−0.0786 (2)	0.0275 (9)	
H12	0.3601	−0.2106	−0.1138	0.033*	
C13	0.36500 (12)	0.0223 (4)	−0.18820 (18)	0.0176 (7)	
C14	0.38140 (15)	−0.0762 (4)	−0.2293 (2)	0.0321 (10)	
H14	0.4059	−0.1298	−0.2147	0.039*	
C15	0.36180 (17)	−0.0955 (4)	−0.2915 (2)	0.0379 (11)	
H15	0.3733	−0.1615	−0.3198	0.045*	
C16	0.32557 (14)	−0.0191 (4)	−0.3126 (2)	0.0278 (9)	
H16	0.3121	−0.0332	−0.3551	0.033*	
C17	0.30910 (13)	0.0775 (4)	−0.27161 (19)	0.0231 (8)	
H17	0.2842	0.1299	−0.2859	0.028*	
C18	0.32898 (12)	0.0983 (4)	−0.20930 (18)	0.0198 (8)	
H18	0.3177	0.1651	−0.1813	0.024*	
C21	0.31812 (12)	0.0474 (4)	0.02718 (18)	0.0189 (7)	
C22	0.27423 (14)	0.0241 (4)	0.00151 (19)	0.0272 (9)	
H22	0.2602	0.0869	−0.0270	0.033*	
C23	0.25146 (15)	−0.0907 (4)	0.0180 (2)	0.0299 (9)	
H23	0.2219	−0.1061	0.0007	0.036*	
C24	0.27177 (15)	−0.1817 (4)	0.0593 (2)	0.0271 (9)	
H24	0.2565	−0.2609	0.0696	0.033*	
C25	0.31401 (15)	−0.1585 (4)	0.0855 (2)	0.0358 (11)	
H25	0.3273	−0.2206	0.1152	0.043*	
C26	0.33761 (14)	−0.0455 (4)	0.0693 (2)	0.0313 (10)	
H26	0.3671	−0.0317	0.0870	0.038*	
C27	0.39002 (12)	0.2361 (4)	0.05878 (17)	0.0180 (7)	
C28	0.43080 (14)	0.2866 (4)	0.0371 (2)	0.0253 (8)	
H28	0.4365	0.2919	−0.0089	0.030*	
C29	0.46323 (15)	0.3293 (4)	0.0834 (2)	0.0308 (9)	
H29	0.4910	0.3643	0.0688	0.037*	
C30	0.45520 (15)	0.3209 (4)	0.1501 (2)	0.0301 (9)	
H30	0.4775	0.3498	0.1814	0.036*	
C31	0.41511 (15)	0.2708 (4)	0.1716 (2)	0.0305 (9)	
H31	0.4099	0.2654	0.2177	0.037*	
C32	0.38215 (14)	0.2280 (4)	0.12689 (19)	0.0278 (9)	
H32	0.3545	0.1936	0.1422	0.033*	
C33	0.30720 (12)	0.3182 (3)	−0.01112 (17)	0.0162 (7)	
C34	0.30652 (13)	0.3962 (4)	−0.0675 (2)	0.0220 (8)	
H34	0.3277	0.3817	−0.1012	0.026*	
C35	0.27452 (14)	0.4961 (4)	−0.0745 (2)	0.0264 (9)	
H35	0.2742	0.5499	−0.1129	0.032*	
C36	0.24337 (14)	0.5172 (4)	−0.0259 (2)	0.0289 (9)	
H36	0.2212	0.5839	−0.0316	0.035*	
C37	0.24440 (13)	0.4413 (4)	0.0312 (2)	0.0284 (9)	
H37	0.2234	0.4576	0.0649	0.034*	
C38	0.27615 (12)	0.3414 (4)	0.03915 (19)	0.0212 (8)	
H38	0.2768	0.2892	0.0782	0.025*	
N1	0.36970 (10)	0.1700 (3)	−0.07309 (14)	0.0184 (6)	
P1	0.39496 (3)	0.05850 (9)	−0.11203 (4)	0.01427 (18)	
P2	0.34805 (3)	0.18839 (9)	−0.00264 (4)	0.01560 (18)	
C41	0.18730 (11)	0.2625 (3)	0.25464 (17)	0.0145 (7)	
C42	0.17620 (13)	0.2292 (4)	0.18920 (19)	0.0203 (8)	
H42	0.1505	0.2672	0.1678	0.024*	
C43	0.20287 (14)	0.1400 (4)	0.1553 (2)	0.0267 (9)	
H43	0.1955	0.1183	0.1107	0.032*	
C44	0.24003 (13)	0.0829 (4)	0.1866 (2)	0.0240 (8)	
H44	0.2580	0.0221	0.1632	0.029*	
C45	0.25116 (12)	0.1138 (4)	0.2517 (2)	0.0220 (8)	
H45	0.2765	0.0738	0.2731	0.026*	
C46	0.22500 (12)	0.2037 (4)	0.28578 (18)	0.0186 (7)	
H46	0.2327	0.2254	0.3303	0.022*	
C47	0.15111 (11)	0.3477 (4)	0.38024 (17)	0.0160 (7)	
C48	0.14568 (13)	0.2155 (4)	0.39693 (19)	0.0213 (8)	
H48	0.1494	0.1495	0.3642	0.026*	
C49	0.13478 (13)	0.1795 (4)	0.46110 (19)	0.0243 (8)	
H49	0.1314	0.0893	0.4724	0.029*	
C50	0.12895 (13)	0.2764 (4)	0.50829 (19)	0.0249 (8)	
H50	0.1216	0.2521	0.5521	0.030*	
C51	0.13368 (13)	0.4089 (4)	0.49246 (19)	0.0220 (8)	
H51	0.1291	0.4744	0.5251	0.026*	
C52	0.14509 (12)	0.4449 (4)	0.42891 (18)	0.0183 (7)	
H52	0.1489	0.5352	0.4181	0.022*	
C53	0.19486 (11)	0.5331 (3)	0.28942 (18)	0.0175 (7)	
C54	0.22588 (12)	0.5664 (4)	0.3396 (2)	0.0218 (8)	
H54	0.2267	0.5176	0.3797	0.026*	
C55	0.25540 (13)	0.6702 (4)	0.3312 (2)	0.0270 (9)	
H55	0.2761	0.6934	0.3658	0.032*	
C56	0.25474 (14)	0.7405 (4)	0.2723 (2)	0.0301 (10)	
H56	0.2753	0.8110	0.2664	0.036*	
C57	0.22425 (14)	0.7084 (4)	0.2223 (2)	0.0267 (9)	
H57	0.2235	0.7581	0.1825	0.032*	
C58	0.19470 (12)	0.6039 (4)	0.22984 (19)	0.0189 (7)	
H58	0.1744	0.5805	0.1947	0.023*	
C61	0.03503 (12)	0.4829 (4)	0.33008 (18)	0.0180 (7)	
C62	0.03190 (12)	0.3542 (4)	0.35411 (19)	0.0204 (8)	
H62	0.0501	0.2865	0.3364	0.024*	
C63	0.00190 (13)	0.3261 (4)	0.40412 (19)	0.0251 (8)	
H63	−0.0006	0.2386	0.4202	0.030*	
C64	−0.02413 (14)	0.4245 (5)	0.4305 (2)	0.0291 (9)	
H64	−0.0445	0.4045	0.4646	0.035*	
C65	−0.02069 (14)	0.5522 (4)	0.4075 (2)	0.0269 (9)	
H65	−0.0387	0.6194	0.4260	0.032*	
C66	0.00901 (13)	0.5831 (4)	0.3575 (2)	0.0242 (8)	
H66	0.0116	0.6711	0.3421	0.029*	
C67	0.03590 (12)	0.4853 (3)	0.18693 (17)	0.0160 (7)	
C68	0.05620 (13)	0.4409 (4)	0.13041 (19)	0.0224 (8)	
H68	0.0880	0.4297	0.1299	0.027*	
C69	0.02988 (14)	0.4124 (4)	0.0739 (2)	0.0265 (9)	
H69	0.0438	0.3811	0.0351	0.032*	
C70	−0.01626 (14)	0.4298 (4)	0.0745 (2)	0.0262 (9)	
H70	−0.0343	0.4084	0.0365	0.031*	
C71	−0.03612 (13)	0.4789 (4)	0.1309 (2)	0.0286 (9)	
H71	−0.0677	0.4937	0.1309	0.034*	
C72	−0.01038 (12)	0.5063 (4)	0.18714 (19)	0.0217 (8)	
H72	−0.0242	0.5393	0.2257	0.026*	
C73	0.08879 (12)	0.6811 (3)	0.26314 (18)	0.0157 (7)	
C74	0.09200 (12)	0.7526 (4)	0.20413 (19)	0.0211 (8)	
H74	0.0786	0.7201	0.1643	0.025*	
C75	0.11520 (13)	0.8723 (4)	0.2046 (2)	0.0243 (8)	
H75	0.1167	0.9228	0.1651	0.029*	
C76	0.13587 (13)	0.9175 (4)	0.2620 (2)	0.0244 (8)	
H76	0.1525	0.9973	0.2614	0.029*	
C77	0.13264 (12)	0.8477 (4)	0.3206 (2)	0.0210 (8)	
H77	0.1470	0.8797	0.3598	0.025*	
C78	0.10834 (12)	0.7309 (4)	0.32183 (19)	0.0198 (8)	
H78	0.1050	0.6849	0.3623	0.024*	
N41	0.11134 (10)	0.4116 (3)	0.25563 (14)	0.0159 (6)	
P41	0.15782 (3)	0.39312 (9)	0.29476 (4)	0.01339 (17)	
P42	0.07035 (3)	0.51324 (9)	0.26050 (4)	0.01424 (18)	

### Atomic displacement parameters (Å^2^)

**Table:**

	*U*^11^	*U*^22^	*U*^33^	*U*^12^	*U*^13^	*U*^23^
I1	0.01942 (12)	0.02321 (13)	0.02771 (14)	−0.00042 (10)	0.00625 (9)	−0.00172 (10)
I2	0.02414 (13)	0.02105 (13)	0.02784 (14)	−0.00082 (10)	0.00618 (10)	0.00332 (10)
C1	0.0161 (17)	0.0210 (18)	0.0129 (16)	−0.0035 (14)	0.0019 (13)	−0.0014 (14)
C2	0.0157 (17)	0.031 (2)	0.0190 (19)	−0.0015 (15)	−0.0007 (14)	−0.0042 (16)
C3	0.0193 (19)	0.046 (3)	0.024 (2)	0.0003 (18)	0.0038 (16)	−0.0007 (19)
C4	0.023 (2)	0.051 (3)	0.020 (2)	−0.0071 (19)	0.0028 (16)	0.0096 (19)
C5	0.034 (2)	0.033 (2)	0.019 (2)	−0.0098 (18)	−0.0063 (16)	0.0097 (17)
C6	0.0183 (18)	0.0261 (19)	0.0171 (18)	−0.0017 (15)	−0.0030 (14)	0.0017 (15)
C7	0.0163 (17)	0.0158 (17)	0.0184 (18)	0.0025 (13)	0.0006 (13)	−0.0007 (14)
C8	0.0188 (18)	0.0200 (18)	0.024 (2)	0.0012 (15)	−0.0031 (15)	0.0003 (15)
C9	0.029 (2)	0.031 (2)	0.024 (2)	0.0043 (17)	−0.0087 (16)	0.0006 (17)
C10	0.037 (2)	0.024 (2)	0.035 (2)	0.0031 (18)	−0.0048 (19)	0.0105 (18)
C11	0.039 (3)	0.024 (2)	0.047 (3)	−0.0070 (19)	−0.014 (2)	0.005 (2)
C12	0.027 (2)	0.025 (2)	0.030 (2)	−0.0044 (17)	−0.0104 (17)	0.0044 (17)
C13	0.0188 (17)	0.0188 (17)	0.0151 (17)	0.0002 (14)	−0.0021 (13)	−0.0032 (14)
C14	0.037 (2)	0.027 (2)	0.031 (2)	0.0196 (18)	−0.0196 (19)	−0.0144 (18)
C15	0.051 (3)	0.032 (2)	0.030 (2)	0.017 (2)	−0.016 (2)	−0.0155 (19)
C16	0.035 (2)	0.026 (2)	0.023 (2)	0.0003 (17)	−0.0132 (17)	−0.0004 (17)
C17	0.0218 (19)	0.0241 (19)	0.023 (2)	0.0003 (15)	−0.0064 (15)	0.0060 (16)
C18	0.0191 (18)	0.0210 (18)	0.0194 (18)	0.0041 (14)	0.0021 (14)	0.0009 (15)
C21	0.0239 (19)	0.0162 (17)	0.0167 (18)	0.0004 (14)	0.0044 (14)	0.0009 (14)
C22	0.038 (2)	0.026 (2)	0.0174 (19)	−0.0094 (18)	−0.0078 (16)	0.0025 (16)
C23	0.038 (2)	0.026 (2)	0.025 (2)	−0.0111 (18)	−0.0047 (18)	−0.0004 (17)
C24	0.040 (2)	0.0174 (19)	0.024 (2)	−0.0059 (17)	0.0103 (17)	−0.0001 (16)
C25	0.036 (2)	0.026 (2)	0.046 (3)	0.0044 (19)	0.009 (2)	0.018 (2)
C26	0.026 (2)	0.029 (2)	0.039 (3)	0.0008 (17)	0.0036 (18)	0.0106 (19)
C27	0.0237 (19)	0.0171 (17)	0.0133 (17)	0.0034 (14)	0.0008 (14)	0.0003 (14)
C28	0.029 (2)	0.026 (2)	0.0206 (19)	−0.0052 (17)	0.0025 (16)	−0.0015 (16)
C29	0.030 (2)	0.030 (2)	0.032 (2)	−0.0066 (18)	−0.0048 (18)	−0.0044 (18)
C30	0.033 (2)	0.030 (2)	0.027 (2)	0.0011 (18)	−0.0104 (17)	−0.0064 (18)
C31	0.037 (2)	0.036 (2)	0.019 (2)	0.0053 (19)	−0.0081 (17)	0.0000 (18)
C32	0.028 (2)	0.039 (2)	0.0166 (19)	0.0016 (18)	0.0032 (16)	0.0030 (17)
C33	0.0170 (17)	0.0163 (17)	0.0153 (17)	−0.0022 (13)	−0.0019 (13)	−0.0026 (14)
C34	0.0228 (19)	0.0147 (17)	0.029 (2)	0.0032 (14)	0.0043 (15)	−0.0015 (15)
C35	0.032 (2)	0.0197 (19)	0.027 (2)	0.0052 (16)	−0.0049 (17)	0.0000 (16)
C36	0.024 (2)	0.022 (2)	0.040 (2)	0.0064 (16)	−0.0063 (18)	−0.0071 (18)
C37	0.022 (2)	0.033 (2)	0.030 (2)	0.0017 (17)	0.0065 (16)	−0.0112 (18)
C38	0.0203 (18)	0.0225 (19)	0.0207 (19)	−0.0007 (15)	0.0019 (15)	−0.0001 (15)
N1	0.0237 (16)	0.0204 (15)	0.0113 (14)	0.0031 (13)	0.0037 (12)	0.0009 (12)
P1	0.0149 (4)	0.0145 (4)	0.0134 (4)	0.0005 (3)	−0.0004 (3)	−0.0008 (3)
P2	0.0184 (4)	0.0151 (4)	0.0134 (4)	0.0010 (3)	0.0019 (3)	0.0003 (3)
C41	0.0110 (15)	0.0156 (16)	0.0171 (17)	−0.0004 (13)	0.0027 (13)	0.0001 (13)
C42	0.0203 (18)	0.0213 (18)	0.0192 (18)	0.0023 (15)	−0.0004 (14)	−0.0020 (15)
C43	0.033 (2)	0.026 (2)	0.021 (2)	0.0062 (17)	−0.0004 (16)	−0.0081 (17)
C44	0.027 (2)	0.0175 (18)	0.028 (2)	0.0026 (15)	0.0098 (16)	−0.0031 (16)
C45	0.0153 (17)	0.0200 (18)	0.031 (2)	0.0028 (14)	0.0016 (15)	0.0006 (16)
C46	0.0191 (18)	0.0187 (17)	0.0181 (18)	−0.0027 (14)	0.0011 (14)	−0.0010 (14)
C47	0.0132 (16)	0.0198 (17)	0.0151 (17)	−0.0007 (13)	0.0003 (13)	0.0023 (14)
C48	0.0254 (19)	0.0190 (18)	0.0195 (19)	0.0005 (15)	−0.0009 (15)	0.0014 (15)
C49	0.025 (2)	0.024 (2)	0.024 (2)	−0.0038 (16)	−0.0006 (16)	0.0048 (16)
C50	0.027 (2)	0.030 (2)	0.0177 (19)	−0.0053 (17)	0.0030 (15)	0.0048 (16)
C51	0.026 (2)	0.0241 (19)	0.0158 (18)	−0.0023 (16)	−0.0004 (15)	−0.0031 (15)
C52	0.0201 (18)	0.0177 (17)	0.0169 (18)	−0.0010 (14)	−0.0015 (14)	0.0001 (14)
C53	0.0143 (16)	0.0148 (17)	0.0236 (19)	−0.0003 (13)	0.0033 (14)	−0.0026 (14)
C54	0.0217 (19)	0.0186 (18)	0.025 (2)	0.0005 (15)	−0.0033 (15)	−0.0003 (15)
C55	0.022 (2)	0.023 (2)	0.036 (2)	−0.0027 (16)	−0.0007 (17)	−0.0083 (18)
C56	0.024 (2)	0.0173 (19)	0.050 (3)	−0.0054 (16)	0.0124 (19)	−0.0062 (18)
C57	0.032 (2)	0.0196 (19)	0.029 (2)	−0.0010 (16)	0.0130 (17)	0.0052 (16)
C58	0.0201 (18)	0.0167 (17)	0.0203 (18)	−0.0003 (14)	0.0068 (14)	0.0007 (14)
C61	0.0163 (17)	0.0194 (18)	0.0184 (18)	−0.0022 (14)	0.0017 (14)	0.0022 (14)
C62	0.0172 (17)	0.0198 (18)	0.0242 (19)	0.0011 (14)	0.0014 (14)	−0.0012 (15)
C63	0.028 (2)	0.027 (2)	0.0207 (19)	−0.0085 (17)	0.0011 (16)	0.0049 (16)
C64	0.025 (2)	0.043 (3)	0.020 (2)	−0.0050 (18)	0.0033 (16)	−0.0023 (18)
C65	0.026 (2)	0.027 (2)	0.028 (2)	−0.0034 (17)	0.0087 (16)	−0.0079 (17)
C66	0.026 (2)	0.0185 (18)	0.028 (2)	0.0015 (15)	0.0056 (16)	−0.0038 (16)
C67	0.0188 (17)	0.0129 (16)	0.0162 (17)	−0.0010 (13)	−0.0027 (13)	0.0018 (13)
C68	0.0233 (19)	0.026 (2)	0.0180 (18)	0.0040 (16)	−0.0029 (15)	−0.0019 (16)
C69	0.038 (2)	0.023 (2)	0.0183 (19)	0.0039 (17)	−0.0049 (17)	0.0002 (16)
C70	0.030 (2)	0.025 (2)	0.023 (2)	−0.0083 (17)	−0.0126 (16)	0.0086 (16)
C71	0.0167 (18)	0.036 (2)	0.033 (2)	−0.0052 (17)	−0.0039 (16)	0.0116 (19)
C72	0.0192 (18)	0.026 (2)	0.0198 (19)	0.0016 (15)	0.0031 (14)	0.0049 (16)
C73	0.0165 (17)	0.0117 (16)	0.0190 (17)	0.0029 (13)	0.0006 (13)	−0.0013 (13)
C74	0.0216 (19)	0.0197 (18)	0.0218 (19)	0.0005 (15)	−0.0034 (15)	−0.0013 (15)
C75	0.029 (2)	0.0186 (18)	0.025 (2)	−0.0003 (16)	0.0047 (16)	0.0048 (16)
C76	0.028 (2)	0.0161 (18)	0.030 (2)	−0.0016 (15)	0.0056 (16)	−0.0026 (16)
C77	0.0196 (18)	0.0177 (18)	0.026 (2)	0.0013 (14)	−0.0022 (15)	−0.0046 (15)
C78	0.0193 (18)	0.0192 (18)	0.0209 (19)	0.0049 (14)	−0.0013 (14)	0.0021 (15)
N41	0.0173 (15)	0.0158 (14)	0.0145 (14)	0.0011 (12)	−0.0007 (11)	−0.0003 (12)
P41	0.0143 (4)	0.0124 (4)	0.0134 (4)	−0.0002 (3)	0.0000 (3)	0.0003 (3)
P42	0.0140 (4)	0.0143 (4)	0.0144 (4)	−0.0001 (3)	0.0009 (3)	0.0005 (3)

### Geometric parameters (Å, °)

**Table:**

C1—C6	1.383 (5)	C41—C42	1.397 (5)
C1—C2	1.403 (5)	C41—C46	1.405 (5)
C1—P1	1.805 (4)	C41—P41	1.793 (4)
C2—C3	1.394 (5)	C42—C43	1.393 (5)
C2—H2	0.95	C42—H42	0.95
C3—C4	1.372 (6)	C43—C44	1.385 (6)
C3—H3	0.95	C43—H43	0.95
C4—C5	1.384 (6)	C44—C45	1.387 (6)
C4—H4	0.95	C44—H44	0.95
C5—C6	1.397 (5)	C45—C46	1.391 (5)
C5—H5	0.95	C45—H45	0.95
C6—H6	0.95	C46—H46	0.95
C7—C12	1.398 (5)	C47—C48	1.395 (5)
C7—C8	1.402 (5)	C47—C52	1.408 (5)
C7—P1	1.795 (4)	C47—P41	1.804 (4)
C8—C9	1.387 (5)	C48—C49	1.393 (5)
C8—H8	0.95	C48—H48	0.95
C9—C10	1.394 (6)	C49—C50	1.385 (6)
C9—H9	0.95	C49—H49	0.95
C10—C11	1.385 (6)	C50—C51	1.391 (5)
C10—H10	0.95	C50—H50	0.95
C11—C12	1.394 (6)	C51—C52	1.385 (5)
C11—H11	0.95	C51—H51	0.95
C12—H12	0.95	C52—H52	0.95
C13—C18	1.379 (5)	C53—C54	1.396 (5)
C13—C14	1.395 (5)	C53—C58	1.403 (5)
C13—P1	1.798 (4)	C53—P41	1.802 (4)
C14—C15	1.387 (6)	C54—C55	1.384 (5)
C14—H14	0.95	C54—H54	0.95
C15—C16	1.385 (6)	C55—C56	1.388 (6)
C15—H15	0.95	C55—H55	0.95
C16—C17	1.381 (6)	C56—C57	1.380 (6)
C16—H16	0.95	C56—H56	0.95
C17—C18	1.394 (5)	C57—C58	1.387 (5)
C17—H17	0.95	C57—H57	0.95
C18—H18	0.95	C58—H58	0.95
C21—C26	1.388 (5)	C61—C62	1.398 (5)
C21—C22	1.411 (5)	C61—C66	1.400 (5)
C21—P2	1.797 (4)	C61—P42	1.800 (4)
C22—C23	1.392 (6)	C62—C63	1.392 (5)
C22—H22	0.95	C62—H62	0.95
C23—C24	1.375 (6)	C63—C64	1.378 (6)
C23—H23	0.95	C63—H63	0.95
C24—C25	1.370 (6)	C64—C65	1.383 (6)
C24—H24	0.95	C64—H64	0.95
C25—C26	1.389 (6)	C65—C66	1.393 (5)
C25—H25	0.95	C65—H65	0.95
C26—H26	0.95	C66—H66	0.95
C27—C28	1.395 (5)	C67—C68	1.380 (5)
C27—C32	1.404 (5)	C67—C72	1.390 (5)
C27—P2	1.804 (4)	C67—P42	1.807 (4)
C28—C29	1.396 (6)	C68—C69	1.398 (5)
C28—H28	0.95	C68—H68	0.95
C29—C30	1.378 (6)	C69—C70	1.381 (6)
C29—H29	0.95	C69—H69	0.95
C30—C31	1.374 (6)	C70—C71	1.388 (6)
C30—H30	0.95	C70—H70	0.95
C31—C32	1.386 (6)	C71—C72	1.383 (6)
C31—H31	0.95	C71—H71	0.95
C32—H32	0.95	C72—H72	0.95
C33—C34	1.388 (5)	C73—C74	1.402 (5)
C33—C38	1.408 (5)	C73—C78	1.403 (5)
C33—P2	1.797 (4)	C73—P42	1.792 (4)
C34—C35	1.395 (5)	C74—C75	1.397 (5)
C34—H34	0.95	C74—H74	0.95
C35—C36	1.382 (6)	C75—C76	1.378 (6)
C35—H35	0.95	C75—H75	0.95
C36—C37	1.387 (6)	C76—C77	1.385 (6)
C36—H36	0.95	C76—H76	0.95
C37—C38	1.392 (5)	C77—C78	1.389 (5)
C37—H37	0.95	C77—H77	0.95
C38—H38	0.95	C78—H78	0.95
N1—P1	1.579 (3)	N41—P41	1.585 (3)
N1—P2	1.587 (3)	N41—P42	1.601 (3)
			
C6—C1—C2	120.7 (3)	C42—C41—C46	119.2 (3)
C6—C1—P1	119.2 (3)	C42—C41—P41	120.1 (3)
C2—C1—P1	119.6 (3)	C46—C41—P41	120.3 (3)
C3—C2—C1	119.2 (4)	C43—C42—C41	119.9 (3)
C3—C2—H2	120.4	C43—C42—H42	120.0
C1—C2—H2	120.4	C41—C42—H42	120.0
C4—C3—C2	120.1 (4)	C44—C43—C42	120.3 (4)
C4—C3—H3	120.0	C44—C43—H43	119.9
C2—C3—H3	120.0	C42—C43—H43	119.9
C3—C4—C5	120.7 (4)	C43—C44—C45	120.5 (4)
C3—C4—H4	119.6	C43—C44—H44	119.8
C5—C4—H4	119.6	C45—C44—H44	119.8
C4—C5—C6	120.2 (4)	C44—C45—C46	119.7 (3)
C4—C5—H5	119.9	C44—C45—H45	120.2
C6—C5—H5	119.9	C46—C45—H45	120.2
C1—C6—C5	119.1 (4)	C45—C46—C41	120.4 (3)
C1—C6—H6	120.5	C45—C46—H46	119.8
C5—C6—H6	120.5	C41—C46—H46	119.8
C12—C7—C8	119.4 (3)	C48—C47—C52	119.3 (3)
C12—C7—P1	122.7 (3)	C48—C47—P41	119.6 (3)
C8—C7—P1	117.9 (3)	C52—C47—P41	120.5 (3)
C9—C8—C7	120.1 (4)	C49—C48—C47	120.6 (4)
C9—C8—H8	119.9	C49—C48—H48	119.7
C7—C8—H8	119.9	C47—C48—H48	119.7
C8—C9—C10	120.1 (4)	C50—C49—C48	119.3 (4)
C8—C9—H9	120.0	C50—C49—H49	120.3
C10—C9—H9	120.0	C48—C49—H49	120.3
C11—C10—C9	120.1 (4)	C49—C50—C51	121.0 (4)
C11—C10—H10	119.9	C49—C50—H50	119.5
C9—C10—H10	119.9	C51—C50—H50	119.5
C10—C11—C12	120.2 (4)	C52—C51—C50	119.8 (4)
C10—C11—H11	119.9	C52—C51—H51	120.1
C12—C11—H11	119.9	C50—C51—H51	120.1
C11—C12—C7	120.1 (4)	C51—C52—C47	120.0 (3)
C11—C12—H12	120.0	C51—C52—H52	120.0
C7—C12—H12	120.0	C47—C52—H52	120.0
C18—C13—C14	119.8 (3)	C54—C53—C58	119.2 (3)
C18—C13—P1	121.0 (3)	C54—C53—P41	122.7 (3)
C14—C13—P1	119.0 (3)	C58—C53—P41	117.8 (3)
C15—C14—C13	119.8 (4)	C55—C54—C53	120.3 (4)
C15—C14—H14	120.1	C55—C54—H54	119.9
C13—C14—H14	120.1	C53—C54—H54	119.9
C16—C15—C14	120.3 (4)	C54—C55—C56	120.1 (4)
C16—C15—H15	119.8	C54—C55—H55	120.0
C14—C15—H15	119.8	C56—C55—H55	120.0
C17—C16—C15	119.8 (4)	C57—C56—C55	120.2 (4)
C17—C16—H16	120.1	C57—C56—H56	119.9
C15—C16—H16	120.1	C55—C56—H56	119.9
C16—C17—C18	120.0 (4)	C56—C57—C58	120.4 (4)
C16—C17—H17	120.0	C56—C57—H57	119.8
C18—C17—H17	120.0	C58—C57—H57	119.8
C13—C18—C17	120.2 (4)	C57—C58—C53	119.8 (4)
C13—C18—H18	119.9	C57—C58—H58	120.1
C17—C18—H18	119.9	C53—C58—H58	120.1
C26—C21—C22	118.7 (4)	C62—C61—C66	120.1 (3)
C26—C21—P2	123.0 (3)	C62—C61—P42	118.4 (3)
C22—C21—P2	118.0 (3)	C66—C61—P42	121.4 (3)
C23—C22—C21	120.1 (4)	C63—C62—C61	119.5 (4)
C23—C22—H22	120.0	C63—C62—H62	120.2
C21—C22—H22	120.0	C61—C62—H62	120.2
C24—C23—C22	120.0 (4)	C64—C63—C62	120.3 (4)
C24—C23—H23	120.0	C64—C63—H63	119.8
C22—C23—H23	120.0	C62—C63—H63	119.8
C25—C24—C23	120.2 (4)	C63—C64—C65	120.3 (4)
C25—C24—H24	119.9	C63—C64—H64	119.8
C23—C24—H24	119.9	C65—C64—H64	119.8
C24—C25—C26	120.8 (4)	C64—C65—C66	120.6 (4)
C24—C25—H25	119.6	C64—C65—H65	119.7
C26—C25—H25	119.6	C66—C65—H65	119.7
C21—C26—C25	120.1 (4)	C65—C66—C61	119.2 (4)
C21—C26—H26	119.9	C65—C66—H66	120.4
C25—C26—H26	119.9	C61—C66—H66	120.4
C28—C27—C32	119.7 (4)	C68—C67—C72	120.2 (3)
C28—C27—P2	118.2 (3)	C68—C67—P42	118.9 (3)
C32—C27—P2	122.1 (3)	C72—C67—P42	120.9 (3)
C27—C28—C29	119.6 (4)	C67—C68—C69	119.8 (4)
C27—C28—H28	120.2	C67—C68—H68	120.1
C29—C28—H28	120.2	C69—C68—H68	120.1
C30—C29—C28	120.3 (4)	C70—C69—C68	120.1 (4)
C30—C29—H29	119.9	C70—C69—H69	119.9
C28—C29—H29	119.9	C68—C69—H69	119.9
C31—C30—C29	120.2 (4)	C69—C70—C71	119.5 (4)
C31—C30—H30	119.9	C69—C70—H70	120.2
C29—C30—H30	119.9	C71—C70—H70	120.2
C30—C31—C32	121.0 (4)	C72—C71—C70	120.7 (4)
C30—C31—H31	119.5	C72—C71—H71	119.7
C32—C31—H31	119.5	C70—C71—H71	119.7
C31—C32—C27	119.3 (4)	C71—C72—C67	119.6 (4)
C31—C32—H32	120.4	C71—C72—H72	120.2
C27—C32—H32	120.4	C67—C72—H72	120.2
C34—C33—C38	120.0 (3)	C74—C73—C78	119.8 (3)
C34—C33—P2	119.6 (3)	C74—C73—P42	119.7 (3)
C38—C33—P2	120.4 (3)	C78—C73—P42	119.2 (3)
C33—C34—C35	119.7 (4)	C75—C74—C73	119.3 (4)
C33—C34—H34	120.1	C75—C74—H74	120.4
C35—C34—H34	120.1	C73—C74—H74	120.4
C36—C35—C34	120.4 (4)	C76—C75—C74	120.3 (4)
C36—C35—H35	119.8	C76—C75—H75	119.8
C34—C35—H35	119.8	C74—C75—H75	119.8
C35—C36—C37	120.2 (4)	C75—C76—C77	120.7 (4)
C35—C36—H36	119.9	C75—C76—H76	119.6
C37—C36—H36	119.9	C77—C76—H76	119.6
C36—C37—C38	120.2 (4)	C76—C77—C78	120.0 (4)
C36—C37—H37	119.9	C76—C77—H77	120.0
C38—C37—H37	119.9	C78—C77—H77	120.0
C37—C38—C33	119.4 (4)	C77—C78—C73	119.8 (3)
C37—C38—H38	120.3	C77—C78—H78	120.1
C33—C38—H38	120.3	C73—C78—H78	120.1
P1—N1—P2	137.6 (2)	P41—N41—P42	134.4 (2)
N1—P1—C7	115.30 (16)	N41—P41—C41	106.87 (16)
N1—P1—C13	110.04 (17)	N41—P41—C53	113.68 (16)
C7—P1—C13	110.25 (17)	C41—P41—C53	104.59 (16)
N1—P1—C1	110.25 (17)	N41—P41—C47	113.20 (16)
C7—P1—C1	106.47 (17)	C41—P41—C47	108.04 (16)
C13—P1—C1	103.87 (17)	C53—P41—C47	109.91 (17)
N1—P2—C33	106.68 (16)	N41—P42—C73	112.57 (16)
N1—P2—C21	114.89 (17)	N41—P42—C61	113.26 (16)
C33—P2—C21	106.23 (17)	C73—P42—C61	108.84 (17)
N1—P2—C27	111.18 (17)	N41—P42—C67	105.34 (16)
C33—P2—C27	108.79 (16)	C73—P42—C67	109.91 (16)
C21—P2—C27	108.80 (17)	C61—P42—C67	106.69 (17)
